# The novel JNK inhibitor AS602801 inhibits cancer stem cells *in vitro* and *in vivo*

**DOI:** 10.18632/oncotarget.8395

**Published:** 2016-03-26

**Authors:** Masashi Okada, Kenta Kuramoto, Hiroyuki Takeda, Hikaru Watarai, Hirotsugu Sakaki, Shizuka Seino, Manabu Seino, Shuhei Suzuki, Chifumi Kitanaka

**Affiliations:** ^1^ Department of Molecular Cancer Science, Yamagata University School of Medicine, Yamagata, Japan; ^2^ Department of Clinical Oncology, Yamagata University School of Medicine, Yamagata, Japan; ^3^ Second Department of Surgery, Yamagata University School of Medicine, Yamagata, Japan; ^4^ Department of Obstetrics and Gynecology, Yamagata University School of Medicine, Yamagata, Japan; ^5^ Research Institute for Promotion of Medical Sciences, Yamagata University Faculty of Medicine, Yamagata, Japan

**Keywords:** cancer initiating cells, c-Jun N-terminal kinase, drug repositioning, serial transplantation assay, xenograft

## Abstract

A phase 2 clinical trial investigating the efficacy and safety of AS602801, a newly developed JNK inhibitor, in the treatment of inflammatory endometriosis is complete. We are now examining whether AS602801 acts against human cancer cells *in vitro* and *in vivo*. *In vitro*, AS602801 exhibited cytotoxicity against both serum-cultured non-stem cancer cells and cancer stem cells derived from human pancreatic cancer, non-small cell lung cancer, ovarian cancer and glioblastoma at concentrations that did not decrease the viability of normal human fibroblasts. AS602801 also inhibited the self-renewal and tumor-initiating capacity of cancer stem cells surviving AS602801 treatment. Cancer stem cells in established xenograft tumors were reduced by systemic administration of AS602801 at a dose and schedule that did not adversely affect the health of the tumor-bearing mice. These findings suggest AS602801 is a promising anti-cancer stem cell agent, and further investigation of the utility of AS602801 in the treatment of cancer seems warranted.

## INTRODUCTION

The c-Jun NH_2_-terminal kinases (JNKs) belong to the mitogen-activated protein (MAP) kinase family. JNK relays, amplifies, and integrates signals from a diverse range of extracellular stimuli, and are thus involved in important cellular processes such as proliferation, apoptosis, and differentiation [1–3]. As such, JNK has been implicated in a number of human diseases, including cancer; the growing body of evidence from animal and human studies suggests a critical role for aberrant activation of JNK in cancer development, and JNK is drawing increasing attention as a promising target of anticancer therapy [4–8].

Although the exact mechanisms by which JNK contributes to cancer development and maintenance remain largely unknown, we have demonstrated that JNK is a key molecule in the maintenance of glioma stem cells and, most importantly, is a viable molecular target for glioma stem cell-directed therapy [9, 10]. Furthermore, we have revealed that this key role of JNK is not unique to glioblastoma, but is shared by other malignancies, such as ovarian, pancreatic, and possibly lung cancers, suggesting that JNK inhibitors like SP600125 could prove effective against different human cancers through the elimination of cancer stem cells [11–13]. However, the safety profile of SP600125 in humans remains unknown.

A phase 2 clinical trial (NCT 01630252) of AS602801, a newly developed, orally-active, ATP-competitive inhibitor of JNK, was recently completed to investigate its efficacy and safety in the treatment of inflammatory endometriosis [14, 15]. Although there has been one study of the immunomodulatory effect of AS602801, the anticancer effects of AS602801 have not been reported to date [15]. Here, we examined the effects of AS602801 on human cancer cells *in vitro* and *in vivo* to determine whether AS602801 might act as a novel anticancer agent.

## RESULTS

### AS602801 shows selective cytotoxic activity against both serum-cultured cancer cells and cancer stem cells *in vitro*

**Figure 1:**
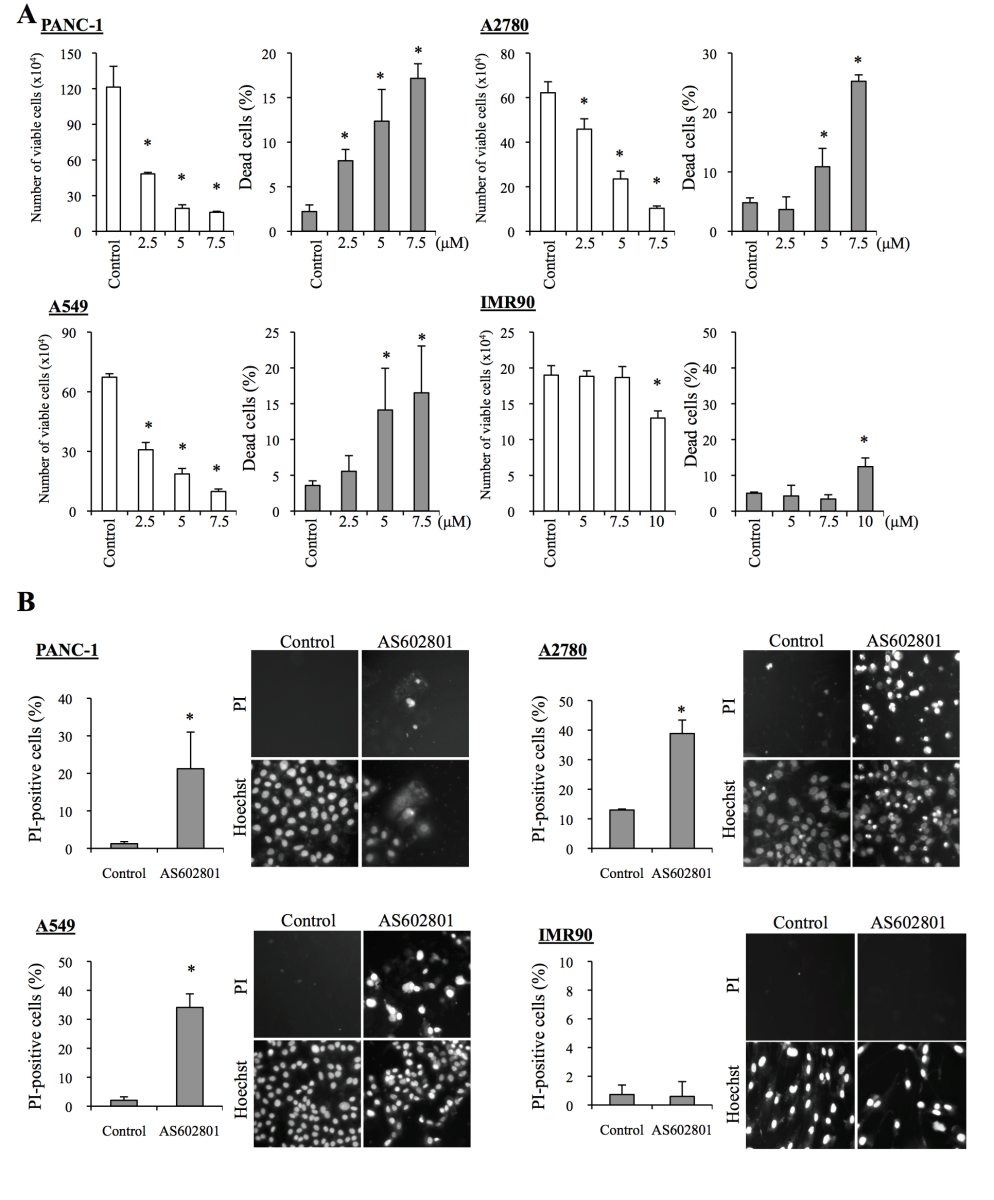
AS602801 induces selective cytotoxicity in serum-cultured human cancer cells **A.** PANC-1, A2780, and A549 human cancer cells and IMR90 human normal fibroblasts were treated without (Control) or with the indicated concentrations of AS602801 for 3 days. The number of viable cells (left panels) and the percentage of dead cells (right panels) were determined using trypan blue as a vital dye. **B.** Cells were subjected to cell death analysis using propidium iodide (PI) as a vital dye after treatment without (Control) or with 7.5 μM AS602801. *Left*, the percentage of PI-positive (dead) cells relative to Hoechst-positive (total) cells was determined. *Right*, representative fluorescence images of PI- (upper rows) and Hoechst-positive cells (lower rows) are shown. Values in the graphs represent the mean + SD from three independent experiments. *P < 0.05.

**Figure 2:**
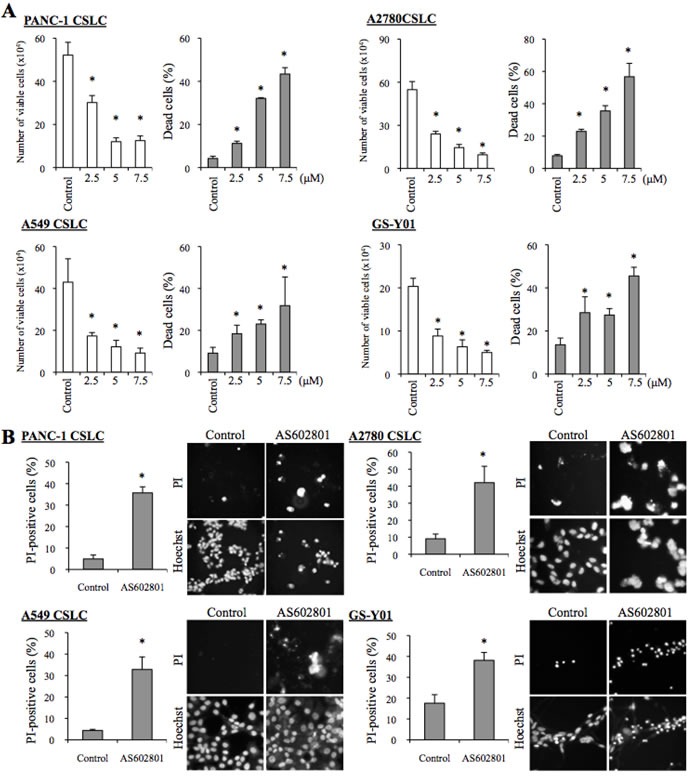
AS602801 has cytotoxic activity against human cancer stem cells **A.** Human cancer stem cell lines (PANC-1 CSLC, A2780 CSC, A549 CSLC, and GS-Y01) were treated without (Control) or with the indicated concentrations of AS602801 for 3 days. Numbers of viable cells (left panels) and percentages of dead cells (right panels) were determined using trypan blue as a vital dye. **B.** Cells were treated without (Control) or with 7.5μM AS602801 for 3 days and then subjected to cell death analysis using propidium iodide (PI) as a vital dye. *Left*, the percentage of PI-positive (dead) cells relative to Hoechst-positive (total) cells was determined. *Right*, representative fluorescence images of PI- (upper rows) and Hoechst-positive (lower rows) cells are shown. Values in the graphs represent the mean + SD from three independent experiments. *P < 0.05.

To determine the range of concentrations of AS602801 that are not toxic to non-neoplastic cells, we first examined the viability of IMR90 normal human fibroblasts after treating them with different concentrations of AS602801. IMR90 cells tolerated up to 10 μM AS602801; we chose a maximum concentration of 7.5 μM for subsequent *in vitro* experiments (Figure 1A). To avoid underestimating cell death, we also analyzed cell death *in situ* using the fluorescent vital dye propidium iodide (PI) instead of trypan blue to reduce the loss of dead cells during the cell collecting procedure. Using this *in situ* method, we confirmed that 7.5 μM AS602801 did not appreciably induce the death of IMR90 cells (Figure 1B). We then examined whether AS602801 had anticancer effects. To this end, we treated three serum-cultured human cancer cell lines (PANC-1, pancreatic cancer; A594, lung cancer; A2780, ovarian cancer) with AS602801 and examined their growth. AS602801 treatment induced cell death and accordingly decreased the number of viable cells in all three cell lines in a dose-dependent manner, suggesting that AS602801 may have selective cytotoxic activity against neoplastic cells (Figure 1A and 1B). We next investigated whether cancer stem cells derived from these cell lines (PANC-1 CSLCs, A549 CSLCs, and A2780 CSLCs) were resistant to AS602801-induced cell death. AS602801 induced cell death in these cells as efficiently as in the original cell lines, suggesting that the cancer stem cell and non-cancer stem cell subpopulations within a cell line are equally sensitive to AS602801 (Figure 2A and 2B). GS-Y01 cells, which are patient-derived glioma stem cells, were also tested to examine whether AS602801 has cytotoxic activity against cells established directly from patient tumor tissues. AS602801 also had cytotoxic activity against GS-Y01 cells (Figure 2A and 2B).

### AS602801 inhibits self-renewal capacity in surviving cancer stem cells

**Figure 3:**
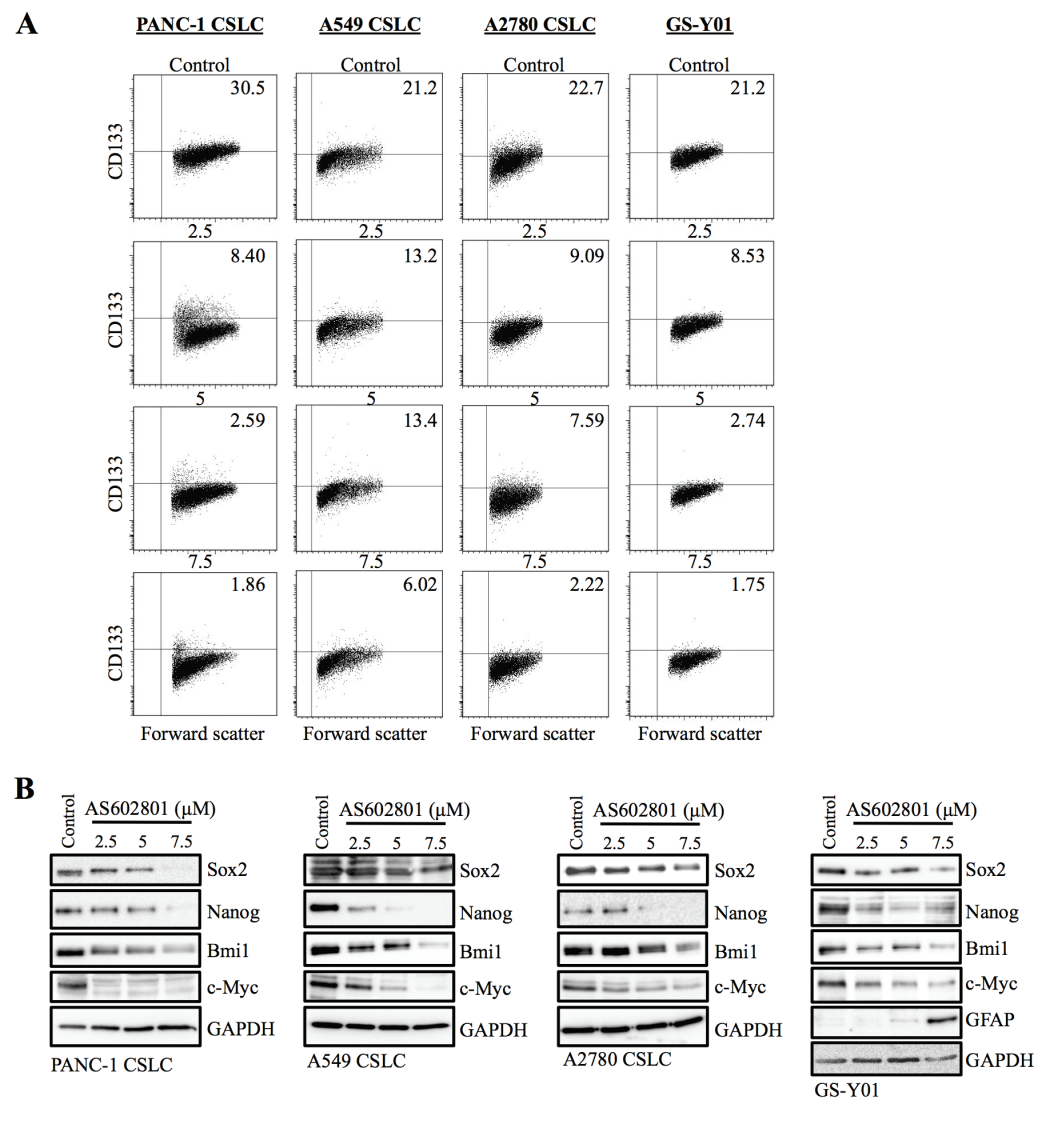
AS602801 treatment causes loss of stem cell marker expression in cancer stem cells **A.** Cells cultured without (Control) or with the indicated concentrations of AS602801 for 6 days were subjected to flow cytometric analysis of the cell-surface expression of CD133. Representative flow cytometric plots together with the percentages of CD133-positive cells are shown. **B.** Cells cultured as described in **A.** were subjected to immunoblot analysis of the indicated protein levels.

**Figure 4:**
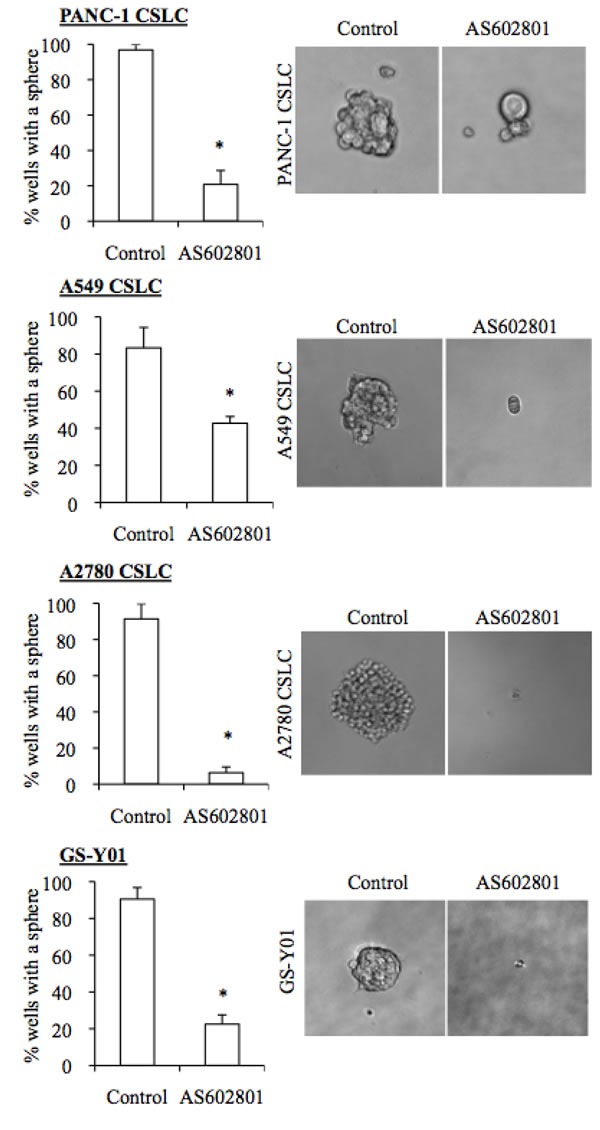
AS602801 induces loss of sphere formation ability in cancer stem cells Cells cultured without (Control) or with 7.5 μM AS602801 for 6 days were subjected to a sphere formation assay in the absence of AS602801. Right panels show photographs of the representative wells. The graphs show the percentage of the wells where a tumor sphere was formed from a single cell. Values in the graphs represent the mean + SD from three independent experiments. *P < 0.05.

Since our previous studies indicated that SP600125 could inhibit the self-renewal capacity of cancer stem cells without causing cell death, we next asked whether self-renewal capacity was also inhibited in cancer stem cells that survived AS602801 treatment. To this end, we first examined the effect of AS602801 treatment on the cell surface expression of CD133, a cancer stem cell marker for various cancer types [16–18]. When the cancer stem cell fraction surviving AS602801 treatment was analyzed by flow cytometry, the proportion of CD133-positive cells decreased in a dose-dependent manner in all cancer stem cell lines examined (Figure 3A). Subsequent analysis revealed that the levels of other stem cell markers, such as Sox2, Nanog, and Bmi1, were decreased similarly to CD133 (Figure 3B). Interestingly, levels of c-Myc, a key pluripotency factor implicated in the maintenance of glioma and other cancer stem cells [19–21], decreased after AS602801 treatment (Figure 3B). In addition to the marker analyses, we examined the effect of AS602801 on the ability of cancer stem cells to self-renew as spheres. When viable cells surviving AS602801 treatment were subjected to a sphere-formation assay in the absence of AS602801, cancer stem cells treated with AS602801 formed fewer spheres compared to control cells (Figure 4). Altogether, these results indicated that, in addition to its cytotoxic activity against cancer stem cells, AS602801 inhibits the self-renewal capacity of cancer stem cells surviving AS602801 treatment.

### AS602801 inhibits tumor-initiating capacity in surviving cancer stem cells

**Figure 5:**
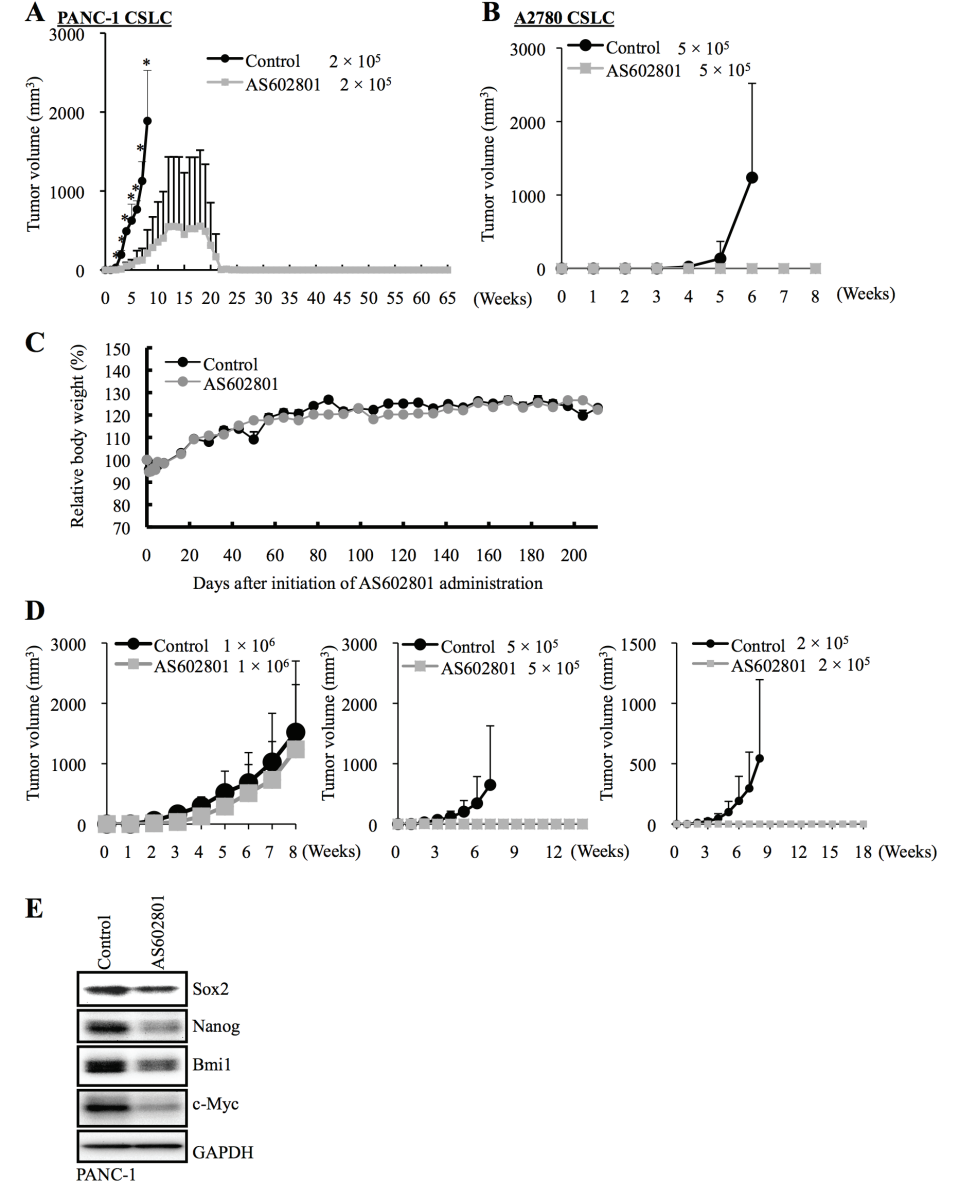
AS602801 treatment inhibits the tumor-initiating capacity of cancer stem cells **A.**, **B.** Mice (3 for each group) were implanted subcutaneously with the indicated number of viable PANC-1 CSLCs **A.** or A2780 CSLCs **B.** that had been treated with or without AS602801 (7.5 μM for PANC-1 CSLCs, 4 μM for A2780 CSLCs) for 6 days. **C.** Two groups of mice (2 mice/group) were given daily intraperitoneal AS602801 injections (40 mg/kg/day) for 10 consecutive days, and their body weight was monitored at the indicated time points. The results are expressed relative to the body weight values recorded at the initial measurement, and represent the mean + SD of the 2 mice in each group. **D.**, **E.** Mice implanted subcutaneously with PANC-1 CSLCs (1 × 10^6^ viable cells) were randomized into 2 treatment groups (4 mice per group) 3 weeks after implantation when the average tumor volume had reached approximately 500 mm^3^, and received a daily intraperitoneal injection of the control vehicle or AS602801 (40 mg/kg/day) for 10 consecutive days. One day after the final drug treatment, the subcutaneous tumors (primary tumors) were excised and dissociated, and the serial dilutions of the dissociated tumor cells were transplanted subcutaneously into new mice. The volumes of the secondary tumors (*n* = 3 for each group) formed by transplantation of the indicated numbers (*Left*: 1 × 10^6^, *Middle*: 5 × 10^5^, *Right*: 2 × 10^5^) of viable cells from the primary tumors treated without (control group) or with AS602801 are presented as mean + SD in the graphs **D.**. Alternatively, the dissociated cells were subjected to immunoblot analysis to measure levels of the indicated proteins **E.**

We next investigated whether cancer stem cells surviving AS602801 treatment also lost tumor-initiating capacity, which is another key feature characterizing cancer stem cells. When an equal number of viable PANC-1 CSLCs was implanted subcutaneously into nude mice after treatment with or without AS602801, cells treated with AS602801 failed to perpetuate tumor growth, whereas untreated cells invariably formed tumors that continued to grow progressively (Figure 5A). Intriguing, all tumors initially formed by AS602801-treated PANC-1 CSLCs eventually regressed spontaneously. Thus, AS602801 deprived the cancer stem cells of their capacity to self-renew indefinitely and support progressive tumor growth, without interfering with their engraftment in nude mice. AS602801 treatment similarly prevented tumor initiation by A2780 CSLCs (Figure 5B). These results demonstrated that AS602801 could inhibit the tumor-initiating capacity of cancer stem cells that survived AS602801 treatment.

### Systemic administration of AS602801 reduces tumor-initiating cancer stem cells *in vivo*

We next sought to determine whether AS602801 could reduce cancer stem cell numbers *in vivo*. Since mice tolerated daily administration of 40 mg/kg AS602801 (Figure 5C), we treated mice bearing subcutaneous tumors formed by implantation of PANC-1 CSLCs (primary tumors) with or without 40 mg/kg AS602801 daily for 10 consecutive days, after which subcutaneous tumors were excised for serial transplantation and stem cell marker analysis. For the serial transplantation analysis, we transplanted different numbers of dissociated cells from the primary tumors subcutaneously into new mice (1 × 10^6^, 5 × 10^5^, 2 × 10^5^) and monitored the mice for the development and growth of secondary tumors. Secondary tumor formation was observed in all but 2 mice (one transplanted with 5 × 10^5^ cells, the other with 2 × 10^5^ cells) transplanted with cells from control-treated tumors, but only in 2 mice transplanted with 1 × 10^6^ cells from AS602801-treated tumors (Figure 5D). Thus, systemic AS602801 treatment reduced the proportion of tumor-initiating cells within the primary tumors. Stem cell marker analysis revealed that Sox2, Nanog, and Bmi1 levels were reduced in primary tumors treated with AS602801 for 10 days (Figure 5E). Similar to the *in vitro* results, c-Myc levels in the primary tumors also decreased after AS602801 treatment (Figure 5E). Collectively, these results demonstrated that AS602801 could reduce numbers of tumor-initiating cancer stem cells when administered systemically to tumor-bearing mice.

## DISCUSSION

Accumulating evidence from clinical and preclinical studies demonstrates that JNK is a critical molecule in the biology of human cancer and an attractive target of cancer therapy [5–8]. Among currently available JNK inhibitors, SP600125 has been used in the overwhelming majority of studies, but the clinical safety profile of this JNK inhibitor remains unknown [22, 23]. Here, we investigated the anticancer effects of a novel JNK inhibitor, AS602801. Our results showed that AS602801 has cytotoxic activity against both serum-cultured (most likely non-stem) cancer cells and cancer stem cells at a concentration that does not decrease the viability of normal human fibroblasts. AS602801 also inhibited the self-renewal and tumor-initiating capacity of cancer stem cells surviving AS602801 treatment. Most importantly, cancer stem cell numbers in established xenograft tumors were reduced by systemic administration of AS602801 at a dose (40 mg/kg) and schedule (peritoneal injection once a day for 10 consecutive days) that did not otherwise affect the health of the tumor-bearing mice. In the clinical trial (NCT01630252) testing its safety and efficacy in the treatment of endometriosis, AS602801 (termed PGL5001 in the trial) was given orally to patients at 320 mg/day (160 mg/day twice a day) for 8 - 20 weeks. Since the human equivalent of the 40 mg/kg/day AS602801 dose given to mice here is approximately 3.3 mg/kg/day when calculated based on the K_m_ of human and mouse [24], our results suggest that AS602801 was effective in the preclinical xenograft model at a clinically relevant dose. Thus, our findings support the use of AS602801 as a therapeutic agent against cancer stem cells.

JNK is receiving increasing attention as an attractive therapeutic target in various pathological conditions, and more JNK inhibitors are being developed [4, 22, 23]. However, developing clinically-applicable JNK inhibitors is not an easy process. For instance, CC-401 and CC-930 are newly developed JNK inhibitors whose effects and safety were successfully demonstrated *in vivo* in a variety of liver and kidney disease models [25–31]. However, a clinical trial to determine the optimal dose of CC-401 in the treatment of high-risk myeloid leukemia was prematurely terminated (NCT00126893). Similarly, CC-930 entered clinical trials to test its safety in the treatment of idiopathic pulmonary fibrosis and discoid lupus erythematosus (NCT01203943, NCT01466725), but the trials were terminated because the benefit/risk profile did not justify continuation of the studies. Thus, publicly available clinical trial results indicate that AS602801 is one of a few JNK inhibitors that were successful in human clinical trials.

**Figure 6:**
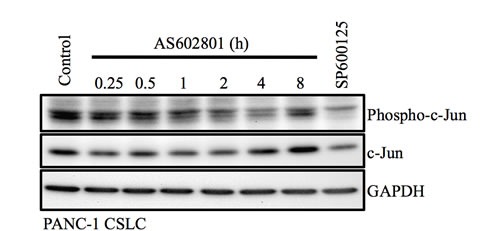
Transient inhibition of c-Jun phosphorylation by AS602801 PANC-1 CSLCs treated with 7.5 μM AS602801 for the indicated times or with 20 μM SP600125 for 1 h were subjected to immunoblot analysis to measure levels of the indicated proteins.

Unexpectedly, we found in this study that inhibition of JNK by AS602801, as indicated by c-Jun phosphorylation status, was apparently transient and short-lived compared to SP600125-induced inhibition (Figure 6) [11, 32]. Although it is possible that phosphorylated c-Jun may not accurately reflect JNK activity, and/or that the transient JNK inhibition was just sufficient for cancer stem cell inhibition, it is also possible that AS602801 has a unique off-target effect(s) that also contributes to the inhibition of cancer stem cells. Our data and previous research indicate that JNK inhibitors do not all act the same; they have different inhibitory profiles depending on JNK isoform, different toxicity/safety profiles, and, presumably, different off-target profiles [4, 22, 23]. Investigations of differences between AS602801 and SP600125 in future studies may therefore shed light on new mechanisms by which JNK acts as a therapeutic target to control cancer stem cells. Additionally, the inhibitory effect of AS602801 on overall tumor growth *in vivo*, in which non-stem cancer cells are important, is yet to be confirmed. Thus, combining AS602801 with chemotherapeutic agents that target non-stem bulk tumor cells might be beneficial in treating cancer.

In conclusion, the present study demonstrates the anticancer effects of AS602801, a drug that has recently been used in a phase 2 clinical trial investigating its efficacy and safety in the treatment of endometriosis. With the available safety information from the clinical trial and our findings that it can reduce cancer stem cell numbers *in vivo*, AS602801 could contribute to the development of curative treatments against human cancers. Future investigations are therefore needed to determine whether this endometriosis drug may also be beneficial in the treatment of cancer.

## MATERIALS AND METHODS

### Antibodies and reagents

Antibodies against Sox2 (#3579), Nanog (#4903), Bmi1 (#6964), GFAP (#3670), phospho-c-Jun (#9261), c-Jun (#9265), and GAPDH (#5174) were purchased from Cell Signaling Technology Inc. (Beverly, MA, USA). Anti-CD133 (W6B3C1) was purchased from Miltenyi Biotech (Bergisch Gladbach, Germany). The antibodies used to detect the expression of Sox2 (MAB2018) and Bmi1 (05-637) in GS-Y01 cells were purchased from R&D Systems Inc. (Minneapolis, MN, USA) and Millipore (Billerica, MA, USA), respectively. AS602801 was purchased from ChemScene (Monmouth Junction, NJ, USA) and was dissolved in DMSO to prepare a 10 mM stock solution.

### Cell culture

The human pancreatic cancer cell line PANC-1 was obtained from the Cell Resource Center for Biomedical Research, Institute of Development, Aging and Cancer, Tohoku University. The human ovarian cancer cell line A2780 was a kind gift from Dr. T. Tsuruo (Institute of Molecular and Cellular Biosciences, University of Tokyo, Japan) and Drs. R.F. Ozols and T.C. Hamilton (the National Institutes of Health, USA) [33]. The human non-small cell lung cancer cell line A549 was obtained from the Riken BioResource Center. These cell lines were maintained in DMEM/F12 medium supplemented with 10% fetal bovine serum (FBS; Sigma) [11–13]. Normal human IMR90 fetal lung fibroblasts were obtained from ATCC and maintained in DMEM supplemented with 10% FBS. Furthermore, the culture medium was supplemented with 100 U/mL penicillin and 100 μg/mL streptomycin. The establishment of human cancer stem cells used in this study (GS-Y01, PANC-1 CSLCs, A2780 CSLCs, and A549 CSLCs) has been described elsewhere [11–13, 34]. These cell lines were maintained under the monolayer stem cell culture condition as previously reported [11–13, 34]. Briefly, cells were cultured on collagen-I-coated dishes (IWAKI, Tokyo, Japan) in the stem cell culture medium (DMEM/F12 medium supplemented with 1% B27 [Gibco-BRL, Carlsbad, CA, USA], 20 ng/mL EGF and FGF2 [Peprotech Inc., Rocky Hill, NJ, USA], D-(+)-glucose [final concentration, 26.2 mM], L-glutamine [final concentration, 4.5 mM], 100 units/mL penicillin, and 100 μg/mL streptomycin). Stem cell culture medium was changed approximately every 3 days, and EGF and FGF2 were added to the culture medium every day. The authenticity of PANC-1 CSLCs, A2780 CSLCs, and A549 CSLCs was verified by the genotyping of short tandem repeat (STR) loci (Bio-Synthesis Inc., Lewisville, TX, USA) followed by comparison to the ATCC STR database (http://www.atcc.org/STR_Database.aspx) for Human Cell Lines. All IMR90 experiments were performed using low passage number (< 8) cells.

### Cytotoxicity assay

Viable and dead cells were identified by their ability and inability to exclude vital dyes, respectively [32, 35]. Briefly, cells were stained with 0.2% trypan blue for 1 min at room temperature (RT), and the number of viable and dead cells was determined using a hemocytometer. The percentage of dead cells was defined as 100 × (number of dead cells/[the number of viable + dead cells]). Alternatively, cells were incubated *in situ* with propidium iodide (PI; 1 μg/mL) and Hoechst 33342 (10 μg/ml) for 10 min at 37°C in a CO_2_ incubator to stain the dead cells and cell nuclei, respectively. Subsequently, the numbers of PI- and Hoechst-positive cells were scored under a fluorescence microscope (CKX41; Olympus, Tokyo, Japan), and the percentage of PI-positive cells (dead cells) relative to Hoechst-positive cells (total cells) was determined.

### Flow cytometric analysis

Flow cytometric analysis was conducted as previously described [11]. Dissociated cells were washed with ice-cold phosphate-buffered saline (PBS), fixed with 4% (w/v) paraformaldehyde for 10 min at RT, and washed again with PBS. The cells were blocked in FCM buffer (0.5% [w/v] bovine serum albumin, 0.1% [w/v] NaN_3_ in PBS) for 1 h, followed by three PBS rinses and further incubation with anti-CD133 antibody in the FCM buffer overnight at 4°C and then with the Alexa Fluor^®^ 488 goat anti-mouse IgG for another 1 h at RT. Gating for single cells was established using forward scatter in the isotype control samples. The isotype control samples were used to establish a gate in the fluorescein isothiocyanate (FITC) channel. Cells showing a signal for CD133 above the gate established by the isotype control were deemed CD133-positive. All flow cytometric analysis experiments were run on the FACSCanto^™^ II Flow Cytometer (BD Biosciences, Franklin Lakes, NJ, USA).

### Immunoblot analysis

Immunoblot analysis was conducted as previously described [11]. Briefly, cells were washed with ice-cold PBS and lysed in RIPA buffer (10 mM Tris-HCl [pH 7.4], 0.1% SDS, 0.1% sodium deoxycholate, 1% NP-40, 150 mM NaCl, 1 mM EDTA, 1.5 mM Na_3_VO_4_, 10 mM NaF, 10 mM sodium pyrophosphate, 10 mM sodium β-glycerophosphate, and 1% protease inhibitor cocktail set III [Sigma]). After centrifugation for 10 min at 14,000 × g at 4°C, the supernatants were recovered as cell lysates, and the protein concentration of the cell lysates was determined by a BCA protein assay kit (Pierce Biotechnology Inc., Rockford, IL, USA). Cell lysates containing equal amounts of protein were separated by SDS-PAGE and transferred to a polyvinylidene difluoride membrane. The membrane was probed with a primary antibody and then with an appropriate HRP-conjugated secondary antibody, according to the protocol recommended by the manufacturer of each antibody. Immunoreactive bands were visualized using Immobilon Western Chemiluminescent HRP Substrate (Millipore).

### Sphere formation assay

The sphere formation assay was performed as previously described [11, 13]. After dissociation, single cells were serially diluted in the stem cell culture medium and seeded into non-coated 96-well plates such that each well contained a single cell. Wells containing a single cell were marked under a phase-contrast microscope on the next day, and 1 week after seeding, the percentage of marked wells with a sphere relative to the total number of marked wells was determined.

### Mouse studies

Mouse xenograft studies were conducted essentially as previously described [11, 36]. For subcutaneous implantation, 6- to 9-week-old male BALB/cAJcl-nu/nu mice (CLEA Japan Inc., Tokyo, Japan) were implanted subcutaneously in the flank region with cells suspended in 200 μL of sterilized PBS under avertin (0.375 g/kg intraperitoneally) anesthesia. After implantation, the recipient mice were monitored for general health status and presence of subcutaneous tumors. For serial transplantation, primary tumors treated as described in the figure legend were excised, and, after a wash in chilled sterile PBS, were transferred into DMEM/F12, minced with scissors, and incubated in Accutase (Sigma) for 30 min at 37°C. After being rinsed with DMEM/F12, the cells were resuspended in DMEM/F12 and filtered through a 70-μm strainer. The single cell suspension was then subcutaneously injected after cell number and viability were determined. Alternatively, the cells were analyzed for the expression of stem cell-related proteins by immunoblotting. For systemic administration of AS602801, the AS602801 stock solution (10 mM in DMSO) was diluted in PBS to prepare 200 μL solutions for each injection. The AS602801 solutions were injected intraperitoneally into nude mice. All control- and AS602801-treated mice received an equal volume of DMSO per body weight (3.6 mL/kg). Tumor volume was determined by measuring tumor diameters (measurement of 2 perpendicular axes of tumors) using a caliper and calculated as 1/2 × (larger diameter) × (smaller diameter)^2^. All animal experiments were performed under a protocol approved by the Animal Research Committee of Yamagata University.

### Statistical analysis

Results are expressed as the mean + standard deviation (SD), and differences were compared using the two-tailed Student's *t*-test. *P*-values < 0.05 were considered statistically significant and are indicated with asterisks in the figures.
